# An optimised system for refolding of human glucose 6-phosphate dehydrogenase

**DOI:** 10.1186/1472-6750-9-19

**Published:** 2009-03-11

**Authors:** Xiao-Tao Wang, Paul C Engel

**Affiliations:** 1 School of Biomolecular and Biomedical Science, Conway Institute, University College Dublin, Belfield, Dublin 4, Ireland

## Abstract

**Background:**

Human glucose 6-phosphate dehydrogenase (G6PD), active in both dimer and tetramer forms, is the key entry enzyme in the pentose phosphate pathway (PPP), providing NADPH for biosynthesis and various other purposes, including protection against oxidative stress in erythrocytes. Accordingly haemolytic disease is a major consequence of G6PD deficiency mutations in man, and many severe disease phenotypes are attributed to G6PD folding problems. Therefore, a robust refolding method with high recovery yield and reproducibility is of particular importance to study those clinical mutant enzymes as well as to shed light generally on the refolding process of large multi-domain proteins.

**Results:**

The effects of different chemical and physical variables on the refolding of human recombinant G6PD have been extensively investigated. L-Arg, NADP^+ ^and DTT are all major positive influences on refolding, and temperature, protein concentration, salt types and other additives also have significant impacts. With the method described here, ~70% enzyme activity could be regained, with good reproducibility, after denaturation with Gdn-HCl, by rapid dilution of the protein, and the refolded enzyme displays kinetic and CD properties indistinguishable from those of the native protein. Refolding under these conditions is relatively slow, taking about 7 days to complete at room temperature even in the presence of cyclophilin A, a peptidylprolyl isomerase reported to increase refolding rates. The refolded protein intermediates shift from dominant monomer to dimer during this process, the gradual emergence of dimer correlating well with the regain of enzyme activity.

**Conclusion:**

L-Arg is the key player in the refolding of human G6PD, preventing the aggregation of folding intermediate, and NADP^+ ^is essential for the folding intermediate to adopt native structure. The refolding protocol can be applied to produce high recovery yield of folded protein with unaltered properties, paving the way for future studies on clinical G6PD mutants with folding defects and providing a useful model system to study the folding process of oligomeric proteins.

## Background

Human glucose 6-phosphate dehydrogenase (G6PD; EC 1.1.1.49) catalyses the first and rate-limiting step in the pentose phosphate pathway. This pathway provides ribose-5-phosphate (R5P) for the synthesis of nucleotides and generates NADPH, both generally for biosynthesis and for more specialised tasks such as protection against oxidative stress in erythrocytes, where G6PD is particularly important as the sole source of NADPH. In connection with this latter role, G6PD deficiency is the most common human enzymopathy, affecting about 400 million people [1,2], and well over 160 different mutations have been determined at the DNA level [3,4]. Recently, the elucidation of the 3-D crystal structure of human G6PD [5,6] has prompted renewed efforts to explain the effects of these mutations. Of the various possible defects [7-10], instability is the prime candidate, since the non-nucleate red blood cells are unable to replace damaged or aged enzyme molecules, and this would explain why haemolytic anaemia is the main manifestation of the deficiency. A folding defect has been suggested as being responsible for the lower stability of clinical G6PD variants [11-13]. The stability of the protein is also thought to be critically dependent on the concentration of NADP^+ ^[14,15], which has been clearly shown to be incorporated into the structure of the folded protein [11,16,17], and impaired binding of this "structural" NADP^+ ^is thought to underlie instability in some clinical mutants. A convenient, robust and reliable refolding method giving high yields for the native enzyme is therefore much needed in order to investigate differences in folding ability between G6PD WT and the mutants.

Of previous attempts to refold human G6PD, the most successful was that of Gomez-Gallego et al. [11], with apparently about 50% recovery (the figure is not explicitly reported). However, in their protocol, protein disulphide isomerase (PDI), the key player, was prepared from bovine liver through time-consuming and tedious procedures with relatively low yield. In addition, GSH and GSSG applied in their method are expensive, and most importantly they did not work in our hands. Therefore, a detailed study on the refolding of human G6PD has now been carried out, leading to an easy and feasible protocol that gives reproducibly high refolding yields, also allowing a thorough survey of the relevant physical parameters and of chemical additives likely to assist the process.

## Results

### Unfolding of human G6PD

Native G6PD (2–4 mg/ml), with free or loosely bound NADP^+ ^previously removed by serial dilution, was denatured by different concentrations of Gdn-HCl in 50 mM Tris-HCl, pH 7.6, at 30°C and kept reduced by the inclusion of 20 mM DTT. The enzyme activity decreased rapidly, and was undetectable after 10 minutes even in 1 M Gdn-HCl. With lower concentrations of Gdn-HCl (e.g. 0.75 M), the denatured protein precipitated, but the resulting pellet could be solubilised at higher concentrations of Gdn-HCl (e.g. 4 M). CD results showed that, in the presence of 4 M Gdn-HCl, after 2 hours all secondary structure was lost (data not shown) and 4 M Gdn-HCl was therefore used routinely to denature the protein in the following experiments.

### Rate of refolding

Under the various conditions explored, the final extent of reactivation varied greatly, but in general the process was slow and required several days in order to achieve completion. In the absence of any beneficial additive the extent of recovery was no more than 1 – 2%. In all the detailed investigations of individual additives below, therefore, the conditions are already partially optimised by the inclusion of one of the other beneficial additives at a constant concentration. In general, the major effect of these additives was on the extent of refolding rather than its speed.

### Effects of L-arginine and DTT

Arginine has long been regarded as a suppressor of protein aggregation and can increase the solubility of aggregation-prone molecules [18-21]. The denatured G6PD protein (1 mg/ml) was diluted quickly into refolding buffer (50 mM Tris, pH 8.0 containing 50 mM NaCl, 200 μM NADP^+^, 10 mM DTT, and 40 μM PEG 3350) to a final concentration of 10 μg/ml with addition of different concentrations of Arg (0–700 mM). The refolding mixture was kept at 25°C and the recovery of enzyme activity was followed. G6PD activity was regained slowly but steadily, reaching a maximum after about 7 days, with 2.6%, 44.3%, 59.4%, 69.5%, 71.1%, 72.2%, 72.1% respectively for 0, 200 mM, 300 mM, 400 mM, 500 mM, 600 mM, and 700 mM Arg added (Table 1), showing that arginine is a highly beneficial additive for refolding. However, when only added to the refolding mixture one day after initiating refolding, Arg was entirely ineffective (data not shown).

**Table 1:** Effects of different concentrations of arginine on the refolding recovery yield.

Added Arg concentration (mM)	0	200	300	400	500	600	700
Refolding yield (%)	2.6 ± 0.1	44.3 ± 3.5	59.4 ± 3.8	69.5 ± 4.1	71.1 ± 4.0	72.2 ± 4.3	72.1 ± 5.1

L-arginine was added into the refolding buffer containing 50 mM NaCl, 200 μM NADP^+^, 10 mM DTT, 40 μM PEG 3350 in 50 mM Tris, pH 8.0 to reach final concentrations of Arg from 0 to 700 mM. The refolding mixture was incubated at 25°C. Activity was monitored over several days and the final regain of G6PD activity is recorded.

Although there are 8 Cys residues in human G6PD, the native protein contains no disulphide bonds. Therefore, maintaining a reducing environment during refolding might be crucial. Without addition of DTT, only 24% of G6PD enzyme activity was recovered even in the presence of high arginine concentrations, as compared with 72% with 10 mM DTT included.

In view of their marked effect, 400 mM Arg and 10 mM DTT were routinely included in the refolding mixtures for most of the remaining experiments and henceforward a refolding buffer containing 50 mM Tris, pH 8.0, 50 mM NaCl, 10 mM DTT, 200 μM NADP^+^, and 400 mM Arg is denoted 'standard refolding buffer'.

### Effects of NADP^+^

**Figure 1:**
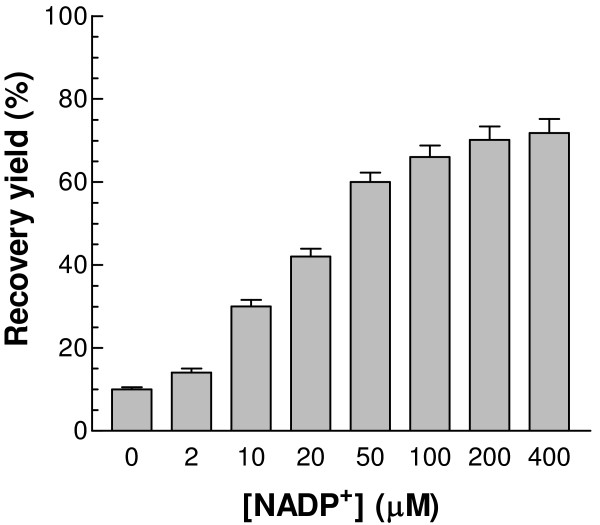
**Effects of different concentrations of NADP^+ ^on the refolding recovery yield**. NADP^+ ^was added into the refolding buffer containing 50 mM NaCl, 400 mM Arg, 10 mM DTT, and 40 μM PEG 3350 in 50 mM Tris, pH 8.0 to reach final NADP^+ ^concentrations from 0 to 400 μM. The refolding mixture was incubated at 25°C and the final regained G6PD activity was followed.

The 3-D structure of human G6PD clearly shows that it has both "structural" and "catalytic" NADP^+ ^binding sites [5,6], with affinities for the nucleotide differing by a factor of 200 [10,22]. It has also been reported that NADP^+ ^is required for monomer hybridization and refolding [11,16,17,23]. Here, NADP^+ ^at different concentrations was added to the refolding buffer (50 mM Tris, pH 8.0, containing 50 mM NaCl, 10 mM DTT, 40 μM PEG 3350, and 400 mM Arg) in order to investigate the effect of the coenzyme on the refolding process. Without added NADP^+^, the recovery yield was only about 10%. As the concentration of NADP^+ ^increased, more enzyme activity could be regained in a concentration dependent manner (Fig. 1). N.B. The lowest coenzyme concentration used in this experiment was in more than 10-fold molar excess over enzyme subunits.

### Effect of cyclophilin A

Based on the 3-D structure of human G6PD [5,6], the *cis/trans *transition of amino acid Pro172 is crucial for the binding of substrate. Since refolding is so slow, the effect of cyclophilin A, a peptidylprolyl cis-trans isomerase reported to facilitate refolding, was investigated. Even with 0.4 μM cyclophilin A in the standard refolding buffer, maximal recovery still took about 7 days. The yield, at about 82%, was about 12% higher than without cyclophilin A. Proline isomerisation would appear not to be a major rate limitation here.

### Effects of other folding additives

**Figure 2:**
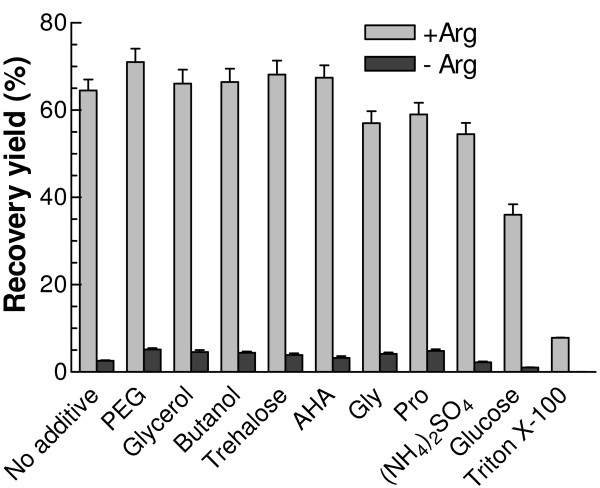
**Effects of different folding additives on the refolding recovery yield**. Respective additive was added into the refolding buffer containing 50 mM NaCl, 200 μM NADP^+^, and 10 mM DTT in 50 mM Tris, pH 8.0 in the presence or absence of 400 mM Arg to final concentration of each additive at 40 μM for PEG 3350, 400 mM for Gly, 400 mM for Pro, 400 mM for glucose, 200 mM for (NH_4_)_2_SO_4_, 200 mM for trehalose, 200 mM for AHA, 5% for glycerol, 0.05% for Triton X-100 and 0.05% for butanol. The refolding mixture was incubated at 25°C and the final regained G6PD activity was recorded.

Various protein folding enhancers, i.e. ammonium sulphate and certain amino acids such as glycine and proline, protein aggregation suppressors, i.e. PEG 3350, Triton X-100, and butanol, and protein stabilizers, i.e. glycerol, trehalose, 6-aminohexanoic acid (AHA) and glucose were added separately in the standard refolding buffer. At the concentrations used, PEG 3350, butanol, glycerol, trehalose and AHA only marginally increased the refolding yield in the presence of Arg (grey bars in Fig. 2), and they were unable to replace Arg in promoting a high refolding yield (black bars in Fig. 2). Glycine and proline offered no further improvement in the refolding process, and glucose and Triton X-100 even appeared to exercise a significant negative effect.

### Effects of salts

**Figure 3:**
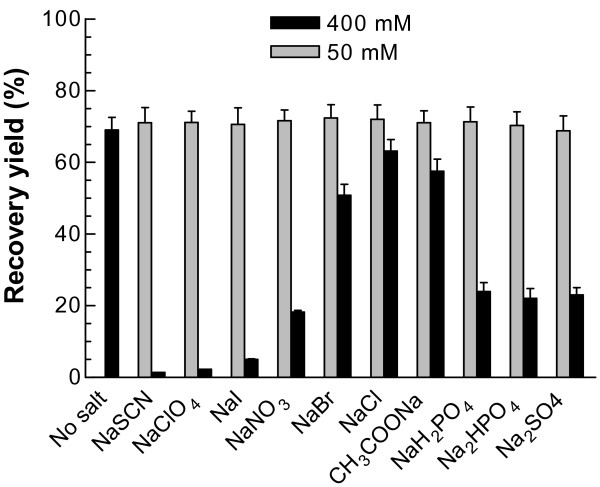
**Effects of different kosmotropic and chaotropic salts on the refolding recovery yield**. Different kosmotropes and chaotropes were added into the refolding buffer containing 400 mM Arg, 200 μM NADP^+^, and 10 mM DTT in 50 mM Tris to final concentrations of each salt at 50 and 400 mM respectively, and pH was adjusted to 8.0. The refolding mixture was incubated at 25°C and the final regained G6PD activity was followed.

Different salts can affect the solubility and stability of proteins. The effects of various kosmotropic and chaotropic salts in the Hofmeister series [24] were therefore investigated. When the added concentration of these different salts was 50 mM in the refolding mixture (50 mM Tris, pH 8.0 containing 400 mM Arg, 200 μM NADP^+^, 10 mM DTT, 40 μM PEG 3350, and 10 μg/ml G6PD), the refolding yields were undistinguishable, at about 70% for all the salts (Fig. 3, grey bars). However, at 400 mM, the effects of the various salts on the refolding were remarkably different, with highest activity regained for NaCl (63%) and NaAc (58%), moderate for the kosmotropic salts Na_2_SO_4 _(23%) and Na_2_HPO_4 _(22%) and the least for the chaotropic salts NaSCN (1.4%) and NaClO_4 _(2.3%) (Fig. 3, black bars).

### Effects of pH and temperature

**Figure 4:**
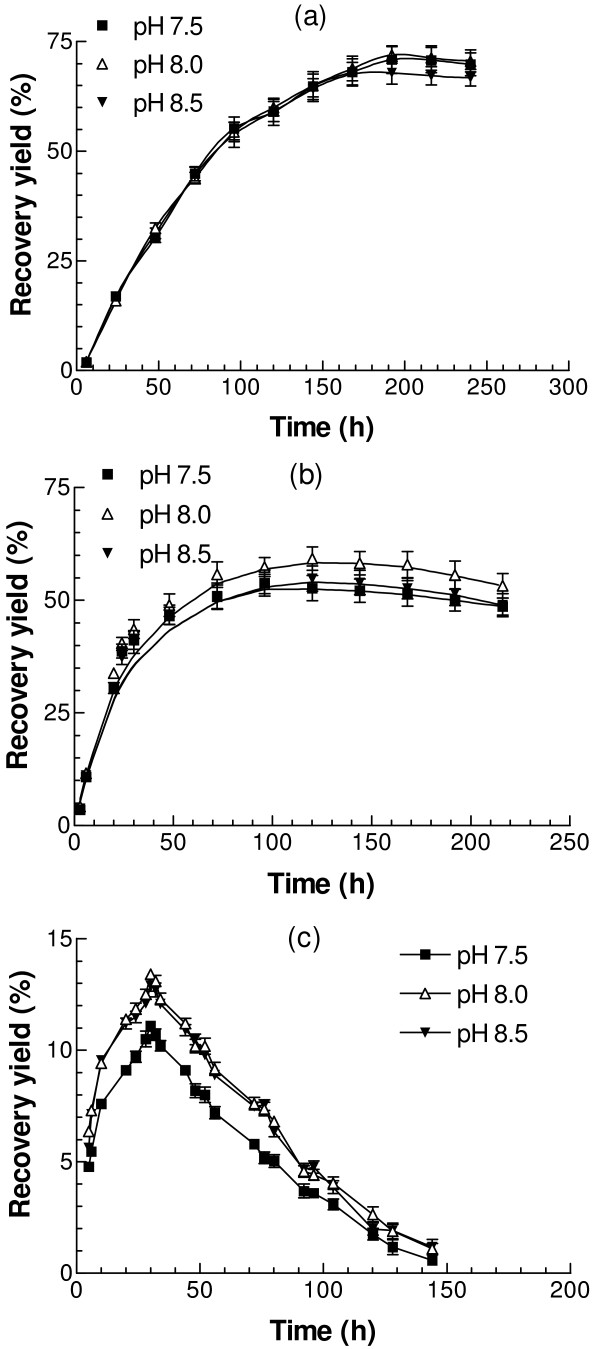
**Effects of temperature and pH on the refolding recovery yield**. Denatured protein was diluted into the refolding buffer containing 50 mM NaCl, 400 mM Arg, 200 μM NADP^+^, and 10 mM DTT in 50 mM Tris with pH ranging from 7.5 to 8.5. The refolding mixture was incubated at (a) 25°C, (b) 30°C, and (c) 37°C, respectively and the regained G6PD activity was followed.

Denatured G6PD protein was diluted into refolding buffer at different pH values ranging from pH 7.5 to pH 8.5 (50 mM Tris, containing 400 mM Arg, 200 μM NADP^+^, 10 mM DTT, 50 mM NaCl, and 40 μM PEG 3350). Refolding was carried out at 25°C, 30°C and 37°C. At 25°C, refolding was relatively slow, taking about one week to reach the maximum activity (Fig. 4). The final refolding yield at pH 7.5 was about 69%, similar to 71% at pH 8.0, and only slightly higher than 65% at pH 8.5, indicating that over this limited range pH is not a critical variable. At 30°C, refolding was faster in the early stages than at 25°C, but the highest activities, achieved after 4–5 days, were only 49% at pH 7.5, 53% at pH 8.0, and 48% at pH 8.5, respectively, considerably lower than at 25°C. Surprisingly, correct refolding was severely suppressed at 37°C, with the highest recovery yield at only 13%.

### Effect of protein concentration

Protein concentration is also a key issue which could affect refolding, on the one hand because G6PD exists in a rapid dimer-tetramer equilibrium, affected by pH and ionic strength [25], and on the other hand because too high a concentration may encourage inappropriate aggregation. In order to optimize the conditions, denatured G6PD was diluted in the standard refolding buffer at final protein concentrations ranging from 5 μg/ml to 50 μg/ml. Recovery yields were 54%, 70%, 56%, and 33% for protein concentrations at 5 μg/ml, 10 μg/ml, 20 μg/ml and 50 μg/ml, respectively. A concentration of ~10 μg/ml thus appears to be optimal.

### Molecular weight of refolded G6PD

**Figure 5:**
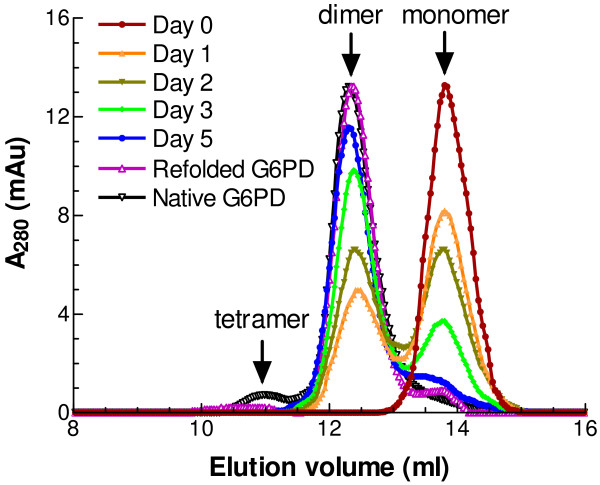
**Gel-elution profile of native G6PD and refolded G6PD by FPLC**. Denatured protein was diluted into the refolding buffer containing 50 mM NaCl, 400 mM Arg, 200 μM NADP^+^, and 10 mM DTT in 50 mM Tris, pH 7.5 at 25°C. Refolded protein samples were withdrawn after incubation for 0, 1, 2, 3, 5, and 7 days. 100 μl samples (around 200 μg/ml) of G6PD refolded protein intermediates and the native protein solutions were loaded on a Superdex 200 HR 10/30 column equilibrated with 0.1 M Tris-HCl, 150 mM NaCl, pH 7.6. Apparent molecular weights of refolded G6PD: 203.6 kDa (10.95 ml), 100.1 kDa (12.36 ml), and 50 kDa (13.78 ml).

Since refolding of G6PD at 25°C is relatively slow, it is possible to follow the changing quaternary structure of the refolding protein. Samples were withdrawn at different time points during refolding and the apparent molecular weights of native, unfolded and refolding G6PD (100 μl at 0.2 mg/ml) were determined by FPLC. The native enzyme gave one major peak and one small minor peak eluting at 12.36 ml and 10.95 ml, corresponding, according to the calibration plot, to M_r _values of 100.1 kDa and 203.6 kDa (Fig. 5). These figures approximate to the values for a dimer (118 kDa) and tetramer (236 kDa). As to the denatured protein, a single dominant peak at 13.78 ml, indicated an M_r _value of 50 kDa. This clearly must be the monomer (59 kDa), although, as with the other two figures, this one is low by about 15%. After one day of refolding, another peak with elution volume at 12.36 ml (dimer) had appeared. As refolding continued, the monomer peak progressively decreased as the dimer peak grew, until finally, after five days, nearly all the refolded protein was dimeric. Thus, for the refolded protein, the main peak with apparent M_r _of around 100 kDa was accompanied by a relative small peak with apparent M_r _of around 200 kDa, the same as the native enzyme (Fig. 5). Another peak, corresponding to monomer (50 kDa), was also clearly seen, indicating that a small amount of denatured protein could not be properly refolded, consistent with the maximal refolding yield at about 70% under current experimental conditions.

### Kinetic parameters of refolded G6PD

A refolded G6PD sample was loaded onto the FPLC column as described above and the eluted protein with M_r _values of 100 kDa and 200 kDa was collected. Detailed kinetic measurements were made for the refolded G6PD for comparison with the native enzyme. The specific activity of the refolded protein was 175 IU/mg, close to 180 IU/mg for the WT enzyme (Table 2). The Dalziel [26] parameters of the refolded enzyme were derived as described for the WT enzyme from primary and secondary plots of the initial rates [9,27]. The *k*_cat _value (1/ϕ_o_) of refolded G6PD (279 s^-1^) was similar to that for native G6PD (275 s^-1^). The *K*_m _values for G6P and NADP^+ ^of refolded G6PD were likewise, within statistical error, unaltered compared with the native enzyme (Table 2).

**Table 2:** Dalziel steady-state kinetic parameters of native and refolded G6PD.

	**Native G6PD**	**Refolded G6PD**
Specific activity (IU/mg)	180 ± 10	175 ± 12
ϕ_o_(s)	0.00366 ± 0.00023	0.00358 ± 0.00031
ϕ_NADP+_(μMs)	0.0259 ± 0.0041	0.0249 ± 0.0037
ϕ_G6P_(μMs)	0.191 ± 0.021	0.196 ± 0.026
ϕ_NADP+G6P_(μM^2^s)	1.61 ± 0.22	1.55 ± 0.18
ϕ_NADP+G6P_/ϕ_NADP+_(μM)	57.98 ± 0.68	62.6 ± 3.4
ϕ_NADP+G6P_/ϕ_G6P_(μM)	7.77 ± 0.68	7.90 ± 0.37
*k*_cat_(s^-1^)	275 ± 18	279 ± 6.8
*K*_mNADP+_(μM)	7.07 ± 1.13	6.94 ± 0.34
*K*_mG6P_(μM)	52 ± 4	54.7 ± 3.2
*k*_cat_/K_mNADP+_(μM^-1 ^s^-1^)	39.7 ± 7.5	40.3 ± 2.5
*k*_cat_/K_mG6P_(μM^-1 ^s^-1^)	5.31 ± 0.69	5.1 ± 0.34

The table shows the four constants of the reciprocal initial-rate equation [26] as estimated for wild-type G6PD and refolded enzyme. The corresponding of *k*_cat _values and the true *K*_m _values for both substrates are also listed. The parameters in each case were obtained from three independent experiments.

### Secondary structure of refolded G6PD

**Figure 6:**
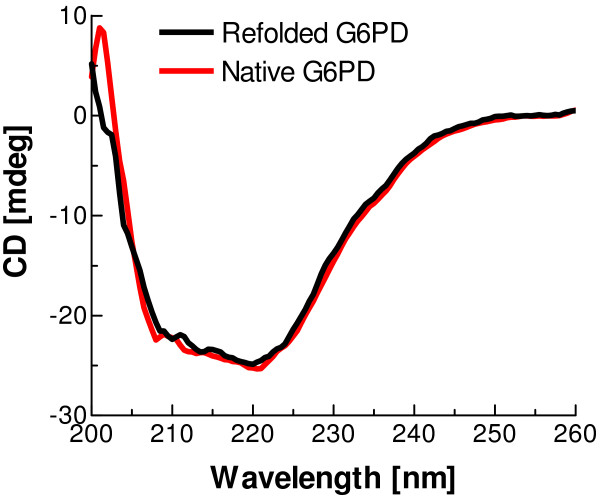
**Far-UV CD spectra of native G6PD and refolded G6PD**. Protein concentration is 0.15 mg/ml and the light path for CD experiment is 2 mm.

The refolded G6PD sample was collected after running an FPLC column as mentioned above. The CD spectra of refolded and native G6PD in the far-UV (200–260 nm) at 25°C were very similar (Fig. 6), indicating that the secondary structure of the refolded enzyme is indistinguishable from that of native G6PD.

## Discussion

The optimised protocol developed here reproducibly gives a high yield of refolded human G6PD and the refolded enzyme has properties indistinguishable from those of native G6PD as judged by steady-state kinetics (Table 2), FPLC studies of quaternary structure (Fig. 5) and far-UV CD study of secondary structure (Fig. 6). Since the refolding process is relatively slow, it should be possible in future to examine the folding intermediates in detail in order to shed further light on the folding pathway of this multi-subunit, multiple-domain protein.

Protein folding involves an intramolecular sequence of events leading to the biological structure, competing with an intermolecular aggregation process. For oligomeric proteins this balance is further complicated by the fact that, whilst aggregation must be avoided, at the same time oligomerisation to give the final active species is also an intermolecular process. In order to improve refolding yields, two types of additive, aggregation suppressors and folding enhancers [28], have been widely used. Aggregation suppressors somewhat destabilize the native structure but prevent aggregation by weakening the intermolecular hydrophobic interactions. Among these suppressors, Arg, containing four types of functional group, i.e. guanidino-, amino-, carboxy-, and methylene-groups, is the most frequently used. While suppressing aggregation and increasing the solubility of denatured proteins [18,20,29,30], it has little effect, in general, on either the thermodynamic stability of folded proteins or the refolding kinetics [21,31,32]. In the present study, the refolding of G6PD was severely impaired without Arg, with only 2.6% recovery yield, compared with 70% in the presence of 500 mM Arg (Table 1). Since Lys, as an alternative basic amino acid, did not increase refolding yield, it seems that the guanidino group of Arg plays a critical role. In addition, since Arg added one day after the initiation of refolding was ineffective, the aggregation that it presumably prevents must occur in the early stages of refolding. Other reputed aggregation suppressors, such as butanol and PEG 3350 increased the refolding yield only slightly (Fig. 2). As to chemical chaperones, trehalose and 6-aminohexanoic acid did not appear to stabilize the renatured G6PD as significantly as previously reported [33]. Moreover, contrary to previous reports that folding enhancers, including sugars, ammonium sulphate and certain amino acids, such as glycine and proline [34-36], can stabilize protein native structure and enhance intermolecular interactions, the results for these additives did not show any positive effects on the refolding yield of G6PD, indicating possibly that the effects of folding enhancers are quite specific to individual proteins.

In this study, it is clearly shown that the refolding yield depends on NADP^+ ^concentration, increasing from 10% to 70% as the NADP^+ ^added to the refolding buffer is increased from 0 to 200 μM (Fig. 1). It should be borne in mind that, although the refolding buffer itself contained no added NADP^+^, the denatured protein will still contain the "structural" NADP^+ ^(1 mol/mol [22]) released during the denaturation process. Whether only the "structural" NADP^+ ^site or both NADP^+ ^binding sites [5,6] are involved in the refolding process needs to be further investigated. Normally, the substrate of an enzyme might help the refolding process, but the study here showed that G6P had no positive effect, even though our kinetic studies [27] would indicate that the sugar phosphate substrate can bind to the folded enzyme in the absence of coenzyme at the active site.

The influence of the salt composition of the refolding solution was also tested. In the Hofmeister series, SCN^-^, ClO_4_^- ^and I^- ^are categorized as chaotropic ions while SO_4_^2- ^and H_2_PO_4_^- ^are kosmotropic. Kosmotropes stabilize protein structure by increasing the amount of water interacting with the protein. In contrast, chaotropes destabilize proteins by breaking down hydrogen bonding and hydrophobic interactions but enhancing solubility. When these salts were present at 400 mM, the differences in the final G6PD recovery yield became substantial. For the kosmotropes, the refolding yields were 23% for Na_2_SO_4 _and 22% for Na_2_HPO_4_. The chaotropes gave extremely low recovery yields, only 1.4% for NaSCN and 2.3% for NaClO_4_. In contrast, salts in the middle of the Hofmeister series gave much higher recovery yields with 63% for NaCl and 58% for CH_3_COONa (Fig. 3). At high concentration, the chaotropes 'salt in' the peptide group, and thus interact much more strongly with the unfolded form of a protein than with its native form. Consequently, they pull the overall equilibrium in the direction of unfolding. On the other hand, high concentrations of kosmotropes strengthen hydrophobic interactions, increasing the risk of aggregation. Therefore, a balance of these two opposing effects is clearly critical for obtaining high recovery yield.

pH, over the range explored, affected the refolding process only to a minor extent (Fig. 4). This is consistent with the accepted view that G6PD is stable from pH 7.5 to 9.0, with maximum activity at pH 8.0 [37]. Temperature, however, affects the refolding process and recovery yield dramatically. At 25°C, refolding was relatively slow, with the maximum 70% activity reached at pH 8.0 after seven days, and this activity could be maintained for a couple of days. At 30°C, the recovery was faster in the early stages as expected, but the highest yield, of about 53% after five days, was less than at 25°C. In contrast, at 37°C, the recovery yield was very low, only about 13% after one day. Also the refolded enzyme was very unstable (Fig. 4). The results suggest that, although the high temperature initially accelerates refolding, it can also destabilize the folded enzyme. Again the balance between two opposing effects becomes quite critical.

The protein concentration in the refolding buffer is also important in determining the extent of refolding. At low protein concentration, the chance of protein molecules interacting to form oligomers is lower. On the other hand, without denaturant, the protein molecules are prone to aggregate at high concentrations. It seems that ~10 μg/ml is the optimal concentration for refolding.

It is interesting to find that the refolding of human G6PD *in vitro *is so slow, taking about seven days to complete even in the presence of peptidylprolyl isomerase. The results here clearly show that the regain of enzyme activity is accompanied by the association of monomer to dimer (Fig. 5). However, the regained activity is not in strict proportion to dimer formation, suggesting that the freshly formed dimer is not fully active, but becomes active after conformational change. These intermediates need to be further investigated in order to understand the relationship between the activity and the quaternary structure of the refolded protein.

Arg, NADP^+ ^and DTT are indispensable components of our optimised refolding buffer. However, in the only previous report of successful refolding of human G6PD [11] the protocol did not include Arg and DTT but employed protein disulphide isomerase and GSH/GSSG. The latter additives were found to be helpful even in the absence of PDI [23]. In the present study, GSH and GSSG did not improve refolding (data not shown). Since there are no disulphide bonds in native human G6PD, PDI is presumably only needed in the refolding mixture if unwanted disulphide bonds are allowed to form. GSSG is thus likely to be a negative factor. The maintenance of a high thiol concentration in our refolding protocol obviates the need for PDI. The preparation of this component required several steps in the previous report and the elimination of this component is clearly advantageous.

In recent decades, attention has been increasingly focused on understanding the protein folding process, not only because misfolding of even a single specific protein *in vivo *can lead to severe disease, but also because large-scale production of active recombinant proteins is in strong demand in pharmaceutical and other industrial fields and often requires refolding of solubilised inclusion bodies. Among these proteins, glyceraldehyde-3-phosphate dehydrogenase (GAPDH) (E.C.1.2.1.12), an important enzyme in glycolysis, has been extensively studied as a model. GAPDH is a tetrameric enzyme of identical subunits containing no disulphide bonds, quite similar to the structural features of G6PD. A systematic study of the variables affecting the refolding of yeast GAPDH by Deal [38], demonstrated that the optimum refolding conditions required nicotinamide-adenine dinucleotide, a reducing environment, and low temperature, in good agreement with the current results for human G6PD. In addition, pH, ionic strength and protein concentration all influenced refolding, as found in this study. Recently, more additives have been found to facilitate the refolding process of rabbit muscle GAPDH, including the *E. coli *trigger factors, crowding agents (PEG 20 K, Dextran 70, and BSA), GroEL, protein disulphide isomerase and α-crystallin [39-43]. In the current study, refolding additives, such as Arg, PEG 3350 and trehalose all assisted refolding. Thus, although refolding is a complicated process, there are significant aspects in common for different proteins.

## Conclusion

It is widely believed that the majority of clinical mutations affecting human G6PD result in destabilisation of the folded native state. It is reasonable to expect this to be reflected not only in the thermal stability of the native protein but also in the ability of the unfolded protein to achieve the folded native state. Our own earlier attempts [13] to compare the folding abilities of normal human G6PD and to two clinical mutants, G6PD_Mahidol _and the very unstable G6PD_Plymouth_, led to results that were qualitatively as expected but nevertheless undermined by the very low levels of recovery. This stimulated the search for a simple and robust procedure that would reproducibly give high refolding yields for the unmutated enzyme. The protocol developed and assessed here reliably delivers upwards of 70% recovery of refolded enzyme with identical characteristics to the native protein. Now that this has been achieved, we believe that this protocol should be useful in the assessment and classification of clinical mutations. We are currently applying the method to several such cases.

## Methods

### Materials

Coenzymes, NADP^+ ^(>98% purity) and NADPH (>98% purity) were supplied by Apollo Scientific Ltd (UK), 2'5' ADP-Sepharose 4B was from GE Healthcare, and the FPLC column, Superdex 200 (10 × 30 cm), was purchased from Pharmacia (now GE Healthcare). Glucose 6-phosphate, guanidinium hydrochloride (Gdn-HCl), L-Arginine monohydrochloride, dithiothreitol (DTT), trehalose, 6-aminohexanoic acid (AHA), polyethylene glycol (MW 3350), Trizma base, cyclophilin A, and other salts such as Na_2_SO_4_, Na_2_HPO_4_, NaH_2_PO_4_, CH_3_COONa, NaI, NaBr, NaClO_4_, NaSCN, etc. were all from Sigma and of reagent grade or better. Spectrophotometric and fluorimetric measurements were made throughout with a Cary Bio50 spectrophotometer and a Hitachi F-4500 fluorimeter. Protein secondary structure was examined with a J810 spectropolarimeter from JASCO (UK).

### Cloning, expression and purification of recombinant human G6PD

Recombinant wild-type human G6PD, over-expressed, purified and stored as previously described [9,27], showed a specific activity of 180 IU/mg. The buffer throughout purification and subsequent procedures was 0.1 M Tris-HCl, pH 7.6, with 5 mM EDTA. 75 μM NADP^+ ^was added to the buffer, both to achieve elution from the affinity column and also to ensure long-term stability of the purified enzyme. For the unfolding experiments, excess NADP^+ ^was rapidly removed by repeated centrifugal filtration through a Centricon YM-50 cone and dilution in fresh buffer.

### Unfolding of recombinant human G6PD

In the unfolding experiments, 8 M Gdn-HCl, dissolved in 50 mM Tris-HCl, pH 7.6, was added to native G6PD to give a final protein concentration of 1 mg/ml and denaturant concentrations from 1 to 6 M, and 20 mM DTT was also included in the mixture to maintain reducing conditions. The mixture was kept at 30°C for two hours in order to denature the protein completely. The extent of denaturation was assessed by spectropolarimetry.

### Refolding of recombinant human G6PD

In the refolding experiments, the reduced and denatured protein was rapidly diluted into the refolding buffer, with addition of different additives at an appropriate concentration, and kept in sealed tubes at various temperatures. The enzyme activity was measured at intervals over periods of up to 10 days, until it ceased to increase, by fluorescence assay, following the increase in the production of NADPH according to WHO guidelines [37]. Excitation and emission wavelengths were 340 nm and 450 nm respectively, with 10 nm band-widths for both light paths. The calculated final refolding recovery yield was based on the ratio of enzyme activity regained against the enzyme activity before denaturation. The effects of various additives and the impacts of pH and temperature on the refolding process were investigated, and the influence of different concentrations of arginine, NADP^+^, salts and G6PD protein concentration was examined in detail.

### FPLC analysis

The molecular weights of G6PD native and refolded proteins were determined by running on an FPLC column Superdex 200 HR 10/30 equilibrated with 0.1 M Tris-HCl, 150 mM NaCl, pH 7.6 and calibrated with bovine erythrocyte carbonic anhydrase (29 kDa), bovine serum albumin (66 kDa), yeast alcohol dehydrogenase (150 kDa), sweet potato β-amylase (200 kDa), and horse spleen apoferritin (443 kDa) from Sigma Aldrich.

### Steady-state kinetic measurement

Steady-state kinetic studies of the refolded protein were carried out fluorimetrically at 25°C as previously reported [9,27]. In brief, reaction mixtures contained 0.01 M MgCl_2 _in 0.1 M Tris-HCl buffer, pH 8.0 with varying amounts of sugar phosphate and coenzyme in a total volume of 1 ml. NADP^+ ^concentrations ranged from 1 to 75 μM and G6P concentrations from 10 to 200 μM. Reaction was initiated by adding enzyme, typically in 10 μl, to give a linear fluorescence increase for at least the first 2 min.

### Circular dichroism analysis

Far-UV CD spectra of native and refolded G6PD were recorded at 25°C on a JASCO J-810 CD spectropolarimeter with a thermal Peltier temperature controller using a 2 mm path length cuvette. Refolded G6PD was loaded onto an FPLC gel filtration column, and eluted protein corresponding to both dimer and tetramer was collected and concentrated using Centricon 50 filtration cones (Millipore, U.S.A.). A desalting column from Pierce Biotechnology was used to decrease salt concentration of the sample. 0.15 mg/ml protein samples of both native and refolded G6PD were applied to explore the secondary structure by following changes in the far-UV region (200–260 nm).

## Abbreviations

G6PD: glucose 6-phosphate dehydrogenase; Arg: L-Arginine monohydrochloride; PDI: protein disulphide isomerase; Gdn-HCl: guanidinium hydrochloride; DTT: dithiothreitol; AHA: 6-aminohexanoic acid; PEG: polyethylene glycol; CD: Circular dichroism.

## Authors' contributions

XTW carried out all experimental work, participated in design of study and drafted the manuscript. PCE conceived the project, supervised the study, and finalized the manuscript. Both authors read and approved the final manuscript.
